# Parliament and Publications

**Published:** 1910-09-10

**Authors:** 


### Parliament and Publications.

### DUTIES OF COUNTY MEDICAL OFFICERS OF HEALTH.

The Local Government Board have made an order prescribing the duties of county medical officers of health. The medical officer is required (1) to inform himself respecting influences affecting injuriously the public health in the county; (2) to report on the isolation hospital accommodation of the county; (3) to communicate to the district medical officers of health any information he may possess as to the danger to health threatening their district; (4) to consult with the district medical officers of health when circumstances render this desirable, and to obtain information from them; (5) to make special reports on any matter appertaining to his duties when required, and to make annual reports on various matters, including the administration of the Sale of Food and Drugs Acts, the Board considering it desirable that the administration of these Acts should, as a rule, be directly under the supervision of the medical officer of health. The Board are prepared to consent to the medical officer of health holding also the office of school medical officer in the county, but they think it is generally undesirable that any other offices should be held by the county medical officer. In regard to the remuneration of the medical officer of health, the Board's experience shows that it is desirable that, in addition to his salary, the officer should be repaid his actual travelling expenses, and that he should be provided with adequate clerical assistance. (Order No. 55,475.)

### BIRTHS, DEATHS, AND MARRIAGES IN SCOTLAND.

The detailed Report regarding the births, deaths, and marriages registered in Scotland during the year 1908 has now been published. The Report shows that the birth-rate is slightly above that of 1907, but is lower than those of all previous years since the institution of national registration, and that is since 1855. The death-rate is lower than the Scottish death-rates of all previous years with the exception of those of 1905 and 1906. The marriage-rate is lower than those of all previous years with five exceptions. The infantile mortality rate of the year was comparatively high; it was higher than the corresponding rates of the preceding three years, and more than the mean of the corresponding rates of the preceding five years. Among causes of death found to be more than usually prevalent during the year there were cerebro-spinal meningitis and measles; a severe epidemic of the former existed in 1907, and that epidemic continued into the first half of 1908. Among causes of death which were less than usually prevalent were enteric fever and tubercle, the death-rates of both being the lowest from those causes yet reported. The death-rate of the year is 16.13, which is rather above the death-rate of the United Kingdom—namely, 15.1. Of the 77,334 deaths registered during the year, and of which the cause was specified, 4,233, or 5.5 per cent., were due to diseases of the nervous system, apoplexies excluded; 13,925, or 18.0 per cent., to diseases of the circulatory system, apoplexies included; 5,871, or 7.6 per cent., to diseases of the respiratory system, pneumonias excluded; 4,915, or 6.4 per cent., to diseases of the digestive system, diarrhœas excluded; 2,748, or 3.6 per cent., to diseases of the urinary system; 220, or 0.3 per cent., to diseases of the generative system; 435, or 0.6 per cent., to diseases and accidents of parturition, puerperal septic infection excluded; and 3,067, or 4.0 per cent., to violent causes. There were 6 deaths from smallpox; 778 from "fevers" (including typhus, enteric, relapsing, cerebro-spinal, and ill-defined fevers). Deaths from measles numbered 2,503; from scarlet fever, 381; from diphtheria, including those certified as due to croup, 748; from whooping-cough, 2,144; from diarrhœa, 3,081. Influenza was certified as the primary cause of death in 419 cases, and as a contributory cause in 815 cases, the total being 1,234. Deaths ascribed to tuberculous disease numbered 9,462. Of the deaths ascribed to violent causes, 109 were due to poison—83 to accidental poisoning and 26 to suicidal poisoning. There were 36 deaths in which anæsthetics were a secondary or contributing cause of death, having been administered for surgical purposes. In 22 of these cases chloroform was the anæsthetic used, in 1 it was ether, in 1 it was stovaine, and in 1 chloroform and ether; in 11 the nature of the anæsthetic was not specified. "Fifty-fourth Detailed Annual Report of the Registrar-General of Births, Deaths, and Marriages in Scotland (Abstracts for 1908." Cd. 5251. Price 2s. 10d.).

